# 

**Published:** 1903-02-21

**Authors:** 


					/the hospital. "The standard oi highest purity."?Lancet. Feb. 21st, 1903.
CADBURY'S ^ ww ^ CADBURY'S
COCOA ^ Jnl
COCOA
ABSOLUTELY PURE.
No. 856. Vol. XXXIII. L-CpS?J Saturday,
Feb. 21st, 1903.
[SUbSCsreePp!035?rm?'] TWOPBNCfl.

				

